# Home visits - central to primary care, tradition or an obligation? A qualitative study

**DOI:** 10.1186/1471-2296-12-24

**Published:** 2011-04-22

**Authors:** Gudrun Theile, Carsten Kruschinski, Marlene Buck, Christiane A Müller, Eva Hummers-Pradier

**Affiliations:** 1 Institute of General Practice and Family Medicine, Hanover Medical School, Carl-Neuberg-Str. 1, 30625 Hannover, Germany

## Abstract

**Background:**

Home visits are claimed to be a central element of primary care. However, the frequency with which home visits are made is declining both internationally and in Germany despite the increase in the number of chronically ill elderly patients. Given this, the question arises as to how to ensure sufficient primary health care for this vulnerable patient group. The aim of this study was to explore German general practitioners' (GPs) attitudes with regard to the feasibility, burden and outlook of continued home visits in German primary care.

**Methods:**

Qualitative semi-structured interviews were carried out with 24 GPs from the city of Hannover, Germany, and its rural surroundings. Data was analysed using qualitative content analysis.

**Results:**

The GPs indicated that they frequently conduct home visits, but not all of them were convinced of their benefit. Most were not really motivated to undertake home visits but some felt obliged to. The basic conditions covering home visits were described as unsatisfactory, in particular with respect to reimbursement and time constraints. House calls for vulnerable, elderly people remained undisputed, whereas visits of a social nature were mostly deleted. Urgent house calls were increasingly delegated to the emergency services. Visits to nursing homes were portrayed as being emotionally distressing. GPs considered good cooperation with nursing staff the key factor to ensure a successful nursing home visit. The GPs wanted to ease their work load while still ensuring quality home care but were unable to suggest how this might be achieved. Better financial compensation was proposed most often. The involvement of specially trained nurses was considered possible, but viewed with resentment.

**Conclusions:**

Home visits are still an integral aspect of primary care in Germany and impose a considerable workload on many practices. Though the existing situation was generally perceived as unsatisfactory, German GPs could not envisage alternatives if asked to consider whether the current arrangements were sustainable in the future. To guarantee an unaltered quality of primary home care, German GPs and health care policy makers should actively initiate a debate on the need for and nature of home visits in the future.

## Background

Home visits are claimed to be a central element of general practice as this represented the primary mode of healthcare delivery by community physicians from the mid-20^th ^century [1]. Today in most European countries and the United States home visits are the exception and are no longer the standard method of health care delivery - although there is diversity between individual general practitioners (GPs) and different countries [2]. Self-employed GPs visit more patients at home than salaried GPs; house calls are less frequent in health care systems where GPs act as gatekeepers and patient lists are maintained [2]. Male GPs make more visits than female doctors, but the latter take more time per visit. In every system there are opponents and supporters of home visiting, e.g. in the United States special house call practices are run. There are however two facts common to all countries and doctors: the number of home visits is continuously declining while the primary target group, namely older, multimorbid people, is growing.

These contradictory findings are the results of several studies published in the last two decades, which have analysed the number of home visits from either quantitative surveys or practice data. They are also true for Germany. However, an analysis conducted at our institute has demonstrated that, despite an overall decreasing frequency of house calls, the number of house visits per home patient has been stable [3]. This finding has supported the assumption of other authors that only house calls of questionable medical importance have been eliminated [2,4].

Despite these considerations, the number of house calls in Germany is still comparatively high with a mean number of 34 visits per GP per week. In Austria, Benelux, and France more than 20 visits were counted on average [2]. At the same time many GPs complain of the heavy workload, the insufficient remuneration and the minimal benefits associated with home visiting. Boerma stated nearly ten years ago that patients prefer home visits much more than their doctors do. As the German primary health care system is dominated by self-employed GPs and is thereby competitive, patients' perspectives undoubtedly account for the German "traditionalism" with respect to home visits. But what are incentives for German GPs to continue or discontinue to make home visits? Who do German GPs visit at home and why? Do German GPs prefer this "delivery service" or can they suggest other models of care? Answers to such questions may be meaningful in the context of considering new potential home care models. Thus, the aim of this study was to explore the attitudes of GPs with regard to the feasibility, burden and outlook for home visits within German primary care in the future.

## Methods

We chose a qualitative approach as our study focussed on subjective GPs' attitudes. As we wished to create a relaxed atmosphere that would allow the GPs to speak freely, we conducted semi-structured interviews at GPs practices or homes.

### Participants

Our intention was to create a purposive sample balanced for the following characteristics: gender, years of occupation, practice location (urban/rural) and practice size (less/more than 2000 patients per quarter). GPs with an entry in the telephone book were contacted initially by telephone and subsequently, if the above mentioned characteristics were compatible, in writing. If a GP was willing to participate, a second call was organised in order to provide more detailed information about the topic and the aim of the study, and to schedule an appointment for the interview. Participants received no remuneration.

### Procedure

The development of the interview guideline was steered by the goal of obtaining opinions, facts and ideas related to home visiting. Potential points of interest and the wording of the questions was discussed by the two main researchers (GT, MB) and finally agreed. The following aspects were included: motivation and organisation of home visits, home visits in nursing homes, procedure and organisation, future perspectives. In addition, the interview started with a provocative warm-up question; a conclusive question at the end allowed a short résumé. (The elaborated interview guideline is depicted in Table 1.) Two pre-tests were conducted to check comprehensibility and fluidity of the guideline. Its final version was condensed into a list of catchwords that were serving as memory hooks representing the detailed questions in order to create a natural conversation. The interviewer (MB) was trained in communication skills. In addition to voice-recordings, handwritten memos were taken in order to document specific or important aspects of each interview. After the interview, GPs filled in a short questionnaire to provide demographic data.

**Table 1:** Interview guideline

Provocative question	Home visits are a core element of general practice. From your point of view: do you agree?
Target group	Who is in need of home visits? When? In what situations?

Motivation	What is different performing home visits compared to consultations in your practice rooms? Do you like to do home visits? How do you feel during home visits?

Organisation	How do you organise home visits? How many home visits do you do per week (or per month)? How long do they take? Area of operation?

Accomplishment	What does a "typical home visit" look like? Is there a "typical home visit"? In your opinion, are there different kinds of home visit and which of them do you perform? Are home visits sufficiently appreciated?

Alternatives	What does the home visit of the future look like to you? What is your opinion of home visits performed by non-medical personnel? How do you judge the idea of preventive home visits?

Home visits in nursing homes	Do you attend home visits in nursing homes? Why? How do you perceive the contact to patients, nursing staff and relatives in this setting? What are the feelings provoked by nursing home visits? What is the nursing home like you attend to? Do you have any personal experience of care homes? How do you imagine your own situation in old age? Are nursing home visits sufficiently appreciated?

Concluding question	What would you suggest to a young colleague concerning (nursing) home visits?

### Analysis

All interviews were digitally recorded and transcribed verbatim by the interviewer herself. Data analysis was conducted independently by two researchers (GT, MB), one of them using ATLAS.ti software, the other coding "by hand". The basis of the analysis was a predefined system of categories, which were generated by the interview guideline. This categorising system was enhanced by codes, which emerged from the text material and were agreed by both researchers after exhaustive discussion and consideration of the additional information from the handwritten notes. In situations where it was not possible to resolve a disagreement, a third researcher was involved until consensus was obtained. By using this iterative approach we refined the initial categorising system and built new codes and subcategories that accounted for themes or aspects of specific topics that had not been considered previously. Furthermore, we analysed some data from the text quantitatively, i.e. number of home visits per week, length of the visits, radius (km) around the practice for home visits as well as demographic data.

## Results

### Participants

A total of 24 GPs agreed to be interviewed, 13 of them male. The median age was 54 years (interquartile range IR 38 - 57 years); the median number of years in private practice was 13 years (4 - 22 years). The GPs came from 14 practices located in Hannover City and 10 in surrounding rural areas. As GPs from group practices were much more likely to agree, single handed practices or group practices consisting of just two partners were underrepresented in the final sample (20.8%). One half of the participating GPs worked in practices with more than 2000 patients per quarter. The mean interview duration was 40 minutes.

### Text Analysis

In the following we describe the theoretical framework, which evolved from the coded text material. The first sub-section is about quantitative data collected during the course of the interviews. Although these numbers cannot be representative, they give an impression of the workload and performance characteristics associated with home visits carried out by the interviewed GPs. Subsequently, different types of home visits, as described by the interviewees, are illustrated; house calls in nursing homes represent a specific category. Lastly, motivational and negative factors are discussed together with ideas for the future of house calls and home care.

### Home visits in numbers

The average home visit conducted by the interviewed GPs lasted 25 min (IQR 17.5 - 30), and took place in the patient's home within a radius of 6.5 (IQR 3-10) km from the practice. The median of the number of home visits per week carried out by a single GP was 6.5 (IQR 3-17.5). For further information regarding the GPs see Table 2. Female GPs conducted considerably fewer home visits than their male colleagues, but they invested more time per visit. The workload resulting from house calls was highest within the group of rural GPs. They required more time per house call and visited more patients per week compared to urban doctors. GPs with more than 20 years of experience conducted fewer home visits than more junior colleagues.

**Table 2:** Number of home visits per GP and length of home visits in median

	Median number of home visits per week (interquartile range)	Median length of home visits (interquartile range)
female GPs	3.0 (IQR 1.5-15.5)	27.5 (IQR 17.5-30.0)

male GPs	7.5 (IQR 5.0 - 20.0)	25.5 (IQR 15.0-37.5)

urbanised area	5.5 (IQR 2.0-20.0)	25.0 (IQR 15.0-30.0)

rural area	7.5 (IQR 3.0-15.5)	27.5 (IQR 17.5-27.5)

20 years or more of occupation	5.0 (IQR 2.0-15.0)	25.0 (IQR 10.0 - 30.0)

5 years or less of occupation	10.0 (IQR 6.5-25.5)	25.0 (IQR 15.0 -30.0)

### Types of home visits

One of the interviewees provided in his own words a very structured differentiation and definition of three types of home visits, which were relevant to most of the other interviews.

"Some of the home visits are of a supportive nature, they actually represent the need for "social interaction" and there isn't a real medical indication. Another class of home visit is to those people who really do need medical care either because they are chronically ill or find it difficult to come to the practice. And then there are those home visits, which are requested due to acute diseases which can be anything from gastrointestinal infection, influenza infection, pneumonia....."

Thus, GPs distinguish between supportive home visits, routine home visits and urgent home visits.

#### Supportive home visits

Home visits of a supportive nature seem to be more important to rural GPs than to those from urban areas. Provincial GPs often perceived themselves not only as medical advisors but also as real companions for their patients. Such traditional professional ethics, which sometimes cross the boundaries of self-abandonment, were rejected by most urban GPs.

"It wasn't my aim to be a minister. In my opinion it's a social problem, which has been cultivated by all of us for decades. But it's not a physician's job to solve this problem."

Business competition in districts with a high density of GPs is apparent. Home visits are reimbursed poorly in Germany and those without any medical indication are avoided.

Most urban GPs deny conducting home visits of a supportive nature, although there is a smooth transition between supportive and routine home visits.

"I have to admit that some time ago, we were more generous with home visits. If there is an old lady with a decubital ulcer or a tumour, then, of course, we still make a routine visit to her. But we don't travel to see all the elderly once a week so that all the elderly in our town get used to a doctor's visit happening every week - that we won't do."

"That is the point: what has changed in the patient? Does he somehow appear different from the last time? Does he have complaints he didn't mention on the telephone? If there's nothing, then there's some small talk, a little social support."

#### Routine home visits

Routine home visits for older chronically ill and increasingly immobile patients are the least challenged visits. All interviewed GPs appreciated the usefulness of routine home visits to detect changes in patients' health status, to control drugs or to achieve an overview of the adequacy of the situation at home.

"Well, you have to listen to how the patients are getting on. You've known them for some time. These are home visits you repeat again and again. You look at how they are doing. You ask after their family, their kids, what they always talk about. You have to know the social environment. You have to look not only if they are alright but also you have to look why he's not on form. Whether he's not got a place in a retirement home or such things for example. Of course you need to talk to them about such things."

#### Urgent home visits

These visits are characterised by an urgent, sometimes immediate need. Most practices have developed an approach to filter the objective requirements related to the patient's concern. Mostly this was done by the GP himself, but in some practices the medical assistants were trained to perform triage on the telephone.

"Yes, you'll assess what awaits you a bit on the telephone. What's good is that we know the patients. For it's either the worsening of a chronic condition, which you can quickly bring under control, or an infection with a high fever or acute diarrhoea. Some call the GP for such illnesses, others don't. In such situations it's possible to establish on the phone, can I help here?"

Only a few of the interviewed GPs left their practice during consultation hours to travel to an extreme case, occasionally after having concurrently informed the emergency services. These GPs argued that no one else knew the medical history of the affected patient as well as they did, and considered themselves the most competent first aider. Most of the other GPs, especially the urban doctors, tended to delegate the real emergency cases -those that could not be delayed- to the emergency services. These GPs rated their competency in real life-threatening situations poorly. They viewed this approach as more reasonable, both with regard to the medical care of the patient and potential economic consequences.

"Well, if I rush out, everything would become so confused - also with the scheduled appointments of other patients and so on. I can't afford that."

In case of febrile exacerbation of an infection or other acute but not life-threatening conditions, most GPs offered their visit after consultation time or during lunch break, and some asked their patients to consider a practice visit regardless of the acute symptoms. Thus, many GPs aimed to reduce the workload associated with urgent house calls.

### Home visits in nursing homes

All of the interviewed GPs conducted visits to nursing homes. However, those working single-handed in a practice limited this type of house call to a very small number, just caring for a few selected patients. Whereas GPs from group practices, in particular with more than two partners, cared for a greater number of patients in nursing homes. The average GP of our interview group visited 20 nursing home patients per week within 1.7 hours. In this context, the physicians reported that they did not see every single patient but conducted mainly chart reviews.

"And I do need two and a half hours for 30 patients (....) so that's five minutes per patient. This is not much. Generally, I don't visit every single patient but only those with acute problems."

Visits in nursing homes were perceived to be quite similar to ward rounds in hospitals. They normally lacked the intimate and confidential atmosphere of a doctor's visit in his patient's home. In most cases, a nurse accompanied the doctor and sometimes family members with their own concerns were present.

Therefore cooperation with the nursing staff was an aspect broadly discussed by many of the interviewed GPs. Most of them described having "trained" the nurses of the regularly visited old people's homes with regard to appropriate telephone calls and preparation for their visits. Experienced nurses seemed to be crucial for successful cooperation, but a shortage of staff and employment of unskilled helpers were frequent problems:

"Most of the nurses are well trained and good natured. For financial reasons staffing is kept to a minimum and this is causing problems."

"There are people who know what to do, but there are also housewives and career changers, who panic and immediately call me or the emergency doctor, when someone has high blood pressure."

The everyday care and examination of a patient's health status was accomplished by the nursing staff, whereas the interviewed GPs often defined their role in nursing homes as "supervisory". Therefore, some of them applied for the right to also oversee the quality standards of the nursing homes, which included the minimum standards concerning staff training.

When reporting on nursing home visits nearly all of the interview partners used remarkably emotional language. No other aspect of the interview guide provoked so many emotive, mainly negative, statements.

"It sometimes reminds me of "One flew over the Cuckoo's Nest."

"It's always about excrement, the whole day, from morning till night."

Nursing homes were described as places of resignation, despair and sadness; they appeared to be sterile, depressing and awful. The interviewed GPs' impression was that nursing home residents were living in forced circumstances and didn't take any notice of each other. A synopsis of all the comments used to describe visits to nursing homes is provided in Table 3. While several GPs mentioned that the nursing homes themselves, i.e. the buildings and facilities had become more pleasant during recent years, only one (female) interviewee was enthusiastic about nursing home visits: "It gives me fulfilment!" She judged these visits, in her role as a GP, as her personal contribution to society. For the other interviewees house calls in nursing homes were an obligation as a result of ethical or financial considerations. Most interview partners did not wish to find themselves in a nursing home in later years.

**Table 3:** Feelings about nursing homes

Nursing homes are a place of...	Nursing homes are...	In nursing homes it is...	Nursing home residents...
...resignation.	...holding institutions.	...sterile.	...don't have a place elsewhere.

...incapacitation.	...barracks for the elderly.	...anonymous.	...feel rejected.

...heteronomy.	...look-outs for death.	...depressing.	...are living in forced circumstances.

...sadness.		...awful.	...gossip about and distinct from each other.

...loneliness.		...gruesome.	...have lost their personality.

...anguish.		...inhumane.	...are unhappy.

...stench.			

...despair.			

...dementia.			

"I mean, if I knew my children didn't want me to live as an invalid in the area or around the corner, then of course I would have to look out a nursing home. But if I had the choice I would prefer to be run over by a bus."

### Motivation for home visits

When asked about their motivation for undertaking home visits, most GPs started with quite vague and general statements, along the lines of: home visits come with the territory.

"It's true, doing home visits is simply part of the job."

"Home visits are obligatory, if you become a general practitioner you have to be prepared to do home visits."

"It's in the nature of the GP's job to be on call."

However, some of the interview partners had good reasons to conduct home visits rather than simply feeling obliged to. For example, the exploration of a patients' home setting and the experience of working in an unfamiliar environment, as opposed to the safe surroundings of their own practice were mentioned in this context.

"And you have a peek on this chaos, a mum with her two kids - there is also a dog bustling around and two cats. Then it's quite clear why these kids have asthma. Father is a smoker. These are things you don't quite realise in your practice, although you can enquire about. But by conducting a home visit you see this at a glance and that's great."

"This is a completely different situation. It's the patient's home. He is the boss and controls the situation. Here in my practice, I do things a certain way and the patient is often very meek. But in his home, he acts completely differently, more independent, and more self-sufficient."

The more positive aspects of performing home visits are summarised in Table 4.

**Table 4:** Positive aspects of home visits

A diversion from the daily routine
Satisfying professional curiosity

Control of medication

Preventing hospitalisation

Immediate help for psychiatric crises

Enhancing the practice's market value

Pleasing the patients

The aforementioned reasons promoting home visiting can not hide the fact that this mode of primary care delivery is not very popular. Just one-third of the interviewed GPs declared that they liked to conduct home visits, the remainder did not.

"It's not the right question [to ask if I like to conduct home visits]. But instead does it make sense to undertake home visits or not? I don't think anybody really likes to do house calls."

When asked for reasons as to why GPs feel reluctant to visit their patient at home pragmatic reasons were initially mentioned.

"Because it is difficult. You are out and about in terrible weather, when it rains or is windy. Most of the people have an awfully untidy apartment. Elderly people always have the windows closed. It's always extremely warm and when you come out you're always sweaty and sticky."

"Then most of the patients - some though are lying in their beds - I have to access in the corner of the living room behind the table in order to get near them. Or I have to move furniture to get to them. I have even visited people in loft beds or in cubby holes, which I have had to crawl into on all fours."

Home visits can put GPs in unpleasant or occasionally even dangerous situations. Moreover, some GPs felt exploited by their patients.

"Yes, I mean, if I'm stressed and have to drive somewhere to an urgent visit, then I sit in my car and complain to myself. My goodness, why can't that guy make a trip to my practice? Doesn't he have any relatives? They all drive to the hairdresser because it is too expensive for the hairdresser to come to them, but the GP can visit."

A couple of GPs referred to the restricted diagnostic options available in the domestic setting, others complained of the poor controllability of consultations in patients' homes. Some GPs even argued home visits promote social isolation because patients were not forced to go outside and meet people on their way to the doctor or in the waiting room. Home visits were perceived as very time consuming. Insufficient reimbursement of such visits in Germany was the most quoted reason for an unwillingness to perform them.

"Well, if I exclusively did home visits, I would go bankrupt. The more I do, the worse it is."

GPs expectations of an adequate payment for house visits were made on the basis of the fees craftsmen charged for their work. Locksmiths, electricians and television engineers were all envied for their hourly wages. Most interviewed physicians proposed a sum at least twice as high as the remuneration currently permitted.

### The future of home visits

Whereas proposals for an adequate financing of home visits were made in sufficient numbers, ideas for the future of home visits rarely emerged. Although most GPs admitted that they conduct home visits reluctantly, they accepted their "duty" without looking for alternatives. Some though proposed the formation of a network of several practices to either reduce the workload of individual GPs or to permit the collective financing of a nurse practitioner who could conduct house calls. Such specially trained nurses were seen as the most favourable alternative to the current situation. In contrast, a district nurse who could also perform house calls was judged very cautiously. GPs suspected that this would introduce redundancy into the existing care arrangements.

"The idea to implement district nurses, I mean, that's fine, but to my eyes it's extremely important to ensure a very close connection to the doctor. The observation I have made is that nursing services, district nurses and all the others don't work together. That means we establish redundant structures. I believe it's very important that medical, nursing and preventive care is centralised."

In addition to concerns about interface problems, a certain fear associated with the establishment of multiple health care providers may also be relevant to statements regarding the future. There were GPs who, while they admitted that they had to get used to the idea of district nurses, nevertheless realised that the involvement of non-medical personnel was an indispensable requirement to cope with the care challenges associated with the anticipated demographic population changes.

Some GPs envisioned the involvement of public volunteers such as socially engaged neighbours to visit isolated elderly people. Few GPs anticipated the complete elimination of home visits. They predicted that in future all patients would be transported to practices somehow, or immediately to the hospital if necessary. This would be co-ordinated from a central office, which would also inform the respective GP about a particular patient's whereabouts and health status.

## Discussion

Home visits are still a component of normal general practice services in Germany. However, GPs are dissatisfied with the conditions associated with conducting them, especially reimbursement, and some doubt the additional value of home visits. A number of house calls are perceived as a "luxury" for demanding patients. Only house calls to vulnerable, elderly people remain undisputed. Home visits in nursing homes are often characterised as emotionally stressful. Despite these issues, the German GPs in our study lacked ideas, if asked about possible future alternatives to the current course of action, both in regard to house calls in patients' homes and to nursing home visits. The suggestions most often discussed included improved financial compensation for home visits and the involvement of nurses specifically trained to take on this duty.

The strength of our study is the good number of interview partners and the robust approach to transcript analysis. Qualitative research excels at the identification of subjective attitudes and experiences. The researcher tries to hold back his own assumptions in favour to record the knowledge of experts who are immersed in the field he wishes to learn more about. In this way, a wide range of relevant information and insights is gathered which may form the basis of further scientific research or influence policy. In this particular study we deliberatively focused on GPs' attitudes while ignoring the perspective of patients or health care policy stakeholders, because we wanted to ascertain the views of those who actually performed the home visits.

Our study also has some limitations. Single-handed practices are underrepresented in our sample, because they were less willing to participate. As the organisational and time constraints associated with home visits for those physicians is high, it can be presumed that they would have been even more critical towards this time consuming mode of primary care. Some of our interview partners from group practices admitted that, had they been in a single practice, they would not be conducting home visits. This is mainly because of the high organisational burden. If a single doctor is on a home visit, who is available to attend to unannounced patients at the practice? We assume that the reluctant willingness of single-handed practices to participate in our interview study corresponds to a reluctance to perform home visits or a desire to perform a smaller number of them. Moreover in future, most German general practices will be group practices because the number of single practices is constantly declining. Given this, the under-representation of single-handed practices in our sample not only seems to be "symptomatic" but also, at least with view to implications for policymakers, acceptable. However, we achieved saturation on all aspects of our interview guide and our findings are consistent with previous research published in this field, indicating a sufficient exploration.

Only the noticeable failure of our respondents to suggest ideas about the future of home visits could have been an indicator that our questions failed to elicit their ideas. It is possibly that focus groups could have been more fruitful with regard to this specific issue, as GPs would have had not only to consider their own experiences but to generate new solutions and to be "creative".

It should be emphasised that although many of our results are similar to those from other countries, it would be inappropriate to generalise. The aim of this study was to uncover the perceptions and issues faced by German GPs regarding home visits.

Of those GPs who participated in our survey, male GPs and those practicing in the countryside in particular routinely made a number of home visits per week. Whereas the frequency of home visits in rural areas seems to depend on the health care tradition of the respective country [2] - e. g. Aylin et al. reported a lower house call rate for the rural population in Wales [5] - the male dominance of those undertaking home visiting is a more "universal phenomenon". The same applies to the fact that female GPs visit fewer patients but spend more time with them [2,6,7]. Our sample of female doctors more frequently worked part-time. But we assume that a different, more patient-centred female working culture is also relevant to the reduced number of patient contacts associated with longer consultation times. Unlike colleagues from the United States [8], none of our interview partners referred to the risk of meeting potential aggressors during home visits. Therefore, at least in Hannover and its surroundings, the fear of attack didn't seem to be a critical issue with regard to the smaller number of home visits made by female GPs. Although most international studies reveal higher home visiting rates for experienced GPs [2] in our sample the younger doctors conducted many more house calls than their older colleagues -probably because German practice owners tend to delegate home visits to their vocational trainees. Svab et al. (2003) reported a similar trend in the Slovenian primary health care system of young residents losing their interest in home visits after completing their training [9].

There is consensus in literature that home visits should be carried out for the old and frail, and critically ill patients [3,4,10-15]. The respondents of our study therefore acted similarly to the majority of their colleagues in Europe, USA and Canada. Nevertheless, house calls for the elderly living at home in Germany were not always of a medical nature and this is also true in other countries [16]. This type of visit was debated by many GPs: whereas some physicians believe this form of interaction not only pleases the patient, but it additionally establishes and improves the reputation for their practice, others propose that such house calls are completely dispensable as their omission would not reduce quality of care. Although supporting data were not available, it can be assumed that a considerable proportion of home visits in Germany are of a supportive social nature. GPs in our interview group as well as in other health care settings [17] responded to this by sharpening their criteria for home visits and "educating" their patients [18], but apparently they still responded to patients' needs [2,4]. Boerma et al. additionally remarked in 1996, that "the strong variation between individual GPs as well as countries in the practice of home visiting suggests a lack of urgency or a need for some of the visits." This "lack of urgency", seems to be true even for genuine emergency visits (those unscheduled house calls, which are made to people with acute conditions). According to our analyses, rural GPs regarded themselves as competent first-aiders because they possessed useful information about patients` history, while urban GPs tended to delegate such emergency house calls more and more to the emergency services. This was not only due to their concerns of dealing with a life-threatening situation when there is an excellent urban emergency ambulance system, but also for economic reasons. Leaving during consultation hours is problematic and if the visit can be delayed into lunchtime or after hours, the question arises if it would not be equally justifiable for the patient to contact a hospital ambulance. Nevertheless all German doctors including specialists working in the ambulant sector are legally bound to conduct home visits. Generally GPs more or less felt obliged to adhere to these regulations. Because of the high density of alternative emergency services, urban GPs seem to be more secure with respect to the consequences of not making home visits. None of the interviewed GPs though referred explicitly to potential liabilities associated with failure to make routine or urgent home visits. GPs may however hesitate to admit that such considerations would affect their decisions.

Although home visiting in the German health care system can be troublesome and may sometimes be a low medical priority, good reasons to perform home visits do exist. Those motives are more personal, accruing from daily work and experience with patients, and are based on a micro, rather than a macro level of health care. First of all, many of our interviewed GPs, similar to other studies [12], regarded home visits as an opportunity to gain additional information about a patient's living conditions, family dynamics and lifestyle. Moreover they additionally obtained a detailed insight into the patient's abilities and their compliance, especially with regard to taking their medication and/or safety issues. Undoubtedly, the direct exploration of a patient's environment yields valuable information, which may improve the quality of care. Some of our respondents concurred with several studies that indicate the potential of home visits to reduce, or at least better inform the suitability of hospitalisation [19,20]. Caplan et al demonstrated that elderly patients with different acute conditions treated at home rather than in hospital were less likely to develop geriatric complications as confusion, bowel or urinary problems. While the overall number of deaths did not differ significantly between the two environments, the patients' and care givers' satisfaction was significantly higher in the "at-home-group" [21]. A recent study from Brazil has also demonstrated the cost effectiveness of home visits in the treatment of alcohol dependent patients [22]. These scientific observations would seem to suggest that home visits deliver an enhanced quality of care and can be cost effective for people suffering from chronic conditions. However, reports describing the effectiveness of home visits by family physicians for a range of conditions encountered in General Practice are still lacking.

Both in our study and in others, the interviewed GPs frequently recognised the positive marketing effect of house calls. One of our interviewees stated that home visits "make patients happy" and there is some research evidence indicating that patients are more dissatisfied with their GP if they undertake fewer home visits [10]. GPs therefore have good reasons for home visiting, even if a strong medical reason does not exist. Boerma et al. showed that in countries where GPs are mainly salaried, the estimated average number of visits per week was much lower than that in countries, such as Germany, where GPs are usually self-employed. This finding suggests that the decision to undertake a home visit can be made more critically in a non-competitive health care system, and that many self-employed GPs will tend to yield to the concrete or assumed wish of their patients - be it for marketing purposes or for liability concerns. Court et al. commented on out of hours requests for GP visits as eliciting an "inappropriate fear of complaint, which is likely to potentiate inappropriate demand for visits" [16]. He concluded that further research should explore how this defensive medical practice could be modified to benefit both patient and practitioner. Nearly 15 years later in Germany, GPs admit to feeling obliged to carry out home visits because they "sell well".

Interestingly, the reason most frequently cited against visiting patients at home is also an economic one: GPs complained about the insufficient remuneration for conducting house calls. In the current German health care system, home visits receive minimal financial compensation and their suggested economic advantages through prevention of hospital admission or psychiatric crises do not have any direct financial impact on the service provided by GPs. Therefore GPs feel that satisfactory financial recompense is one of the most important changes needed with regard to the maintenance of this special form of patient care [17].

In Germany, nursing and residential homes for the elderly do not have their own on-site doctors. Thus as a rule, the GP who has provided care to the elderly patient up to this point will continue to do so after they move into the care home. Home visits to nursing homes differ notably from the "usual" house calls in that they rather resemble ward rounds in hospitals. GPs normally talk much more to the nurses than to their patients. The GPs interviewed in this study commented on the very high emotional burden associated with this sort of home visit. A variety of reasons attracted them to make house calls in nursing homes. These ranged from the relative ease and cost-effectiveness of making visits to multiple patients in one retirement home, to it being professionally ethical to continue to provide care to a long-term patient moving to such an environment; but never has it seemed a real vocation. None of the GPs in our interview group could imagine going to a nursing home themselves in later life. GPs are not specifically trained to work in a care home environment and it is challenging both professionally and psychologically. Therefore, it is not difficult to understand Katz et al. who postulated in 2009 on the creation of a nursing home medicine speciality in the United States [23]. He quotes that the marginal involvement of physicians impedes communication and their integration in nursing home culture, which has a detrimental impact on patient outcomes. Most acute illnesses are managed by telephone, which in the light of the medical complexity of the residents, may not be in their best interests [24]. These observations from the United States are applicable to the German situation given the claims of an interview partner in our study; one GP sought to be involved in the staff management of care homes in order to enhance quality of care of the elderly patients. Consequently we feel that a discussion about specialised nursing home physicians, similar to positions already established in the Netherlands [25], should be conducted more intensely and more open-mindedly than it is currently in Germany.

However, when asked to consider the future of home visits in general, the participants of our study failed to propose any significant ideas. Very few among them had considered reorganising home visits or even home care in the German health care system. The most frequently considered alternative was for home visits to be performed by practice nurses linked to their practice. In fact, the German primary care system is currently offering courses to specially train such nurses (e.g. Versorgungsassistentin in der Hausarztpraxis (VERAH^®^, or Care Assistant in Family Practice) but such supporting personnel are not widely established and reimbursement has limited attractiveness. In this regard Germany is lagging behind other countries, where nurses are medically trained and actively involved in ambulant patient care, even sometimes acting as "substitutes" for doctors. Far less conceivable for the GPs of our interview group, was the engagement of an independent nurse to conduct home visits for multiple, but not necessarily linked general practices. This is despite finding that an initial project to establish whether such an arrangement would be attractive or even essential for undersupplied rural areas in Germany had good acceptance from both patients and the participating GPs [26]. A study from the United Kingdom showed that nurse practitioners could substitute for GPs in out-of-hours-calls without any deterioration in patient satisfaction or clinical management outcomes [27]. One of our respondents could see an abolition of home visits in favour of primary care centres, which patients attend and hospital ambulances would provide transport to if necessary. This is exactly what GPs from an English investigation rated as a possible alternative to out-of-hours visits [17]. Surprisingly, our interview partners did not suggest making more use of technology (e.g. telecommunication, computer guides to diagnosis and treatment) although it is likely to both be used in future home care and change the culture of home visits [28].

The results of our study indicate that a conscious discussion is needed on the pros and cons of home visits in their current form in Germany. GPs willingness to further conduct home visits under the existing conditions is rather small. However there are suggested advantages for primary home care, as economic savings by e.g. avoiding hospital admissions or to ensure health care for housebound elderly. Such presumed advantages must be critically addressed by further health care and economic research. Moreover, patients' perspectives on home visits and those of other stakeholders from the health care system will have to be explored. Another striking point is the GPs' attitude towards nursing home visits. Further research needs to evaluate the current situation in German nursing homes by assessing the attitudes and needs of patients and nurses. Our Institute of General Practice and Family medicine will contribute to this important, under-researched field by conducting a qualitative empirical analysis of interprofessional collaboration and communication in nursing homes. The planned study is funded by the German Federal Ministry of Education and Research. It will include 120 interviews with GPs, nurses, residents and their relatives, and additional direct observations and focus groups involving another 100 individuals from the various stakeholders. In this way we hope to achieve a broad insight into medical care in nursing homes.

What ever change future will bring to the home visit system as it now stands: the quality of primary health care must be maintained while an out-dated system will have to be modernised so that it can deal with the shifting population demographics.

## Conclusions

While home visits in Germany seem to represent a primary care tradition and are rated to have clear advantages with regard to patient care they are nevertheless perceived as an obligation by many GPs. A conscious discussion on the pros and cons of home visits in their current form in Germany seems to be indispensable. Visits to nursing homes are perceived as emotionally stressful. Further research is needed to obtain a broad insight into GPs' (negative) attitudes towards nursing homes and to develop improvement strategies concerning primary home care. If home care by GPs is politically desired or recognised by society as the best way to ensure good medical care particularly for the very old and sick, appropriate financial incentives would effectively guarantee a continuation of this "tradition". To quote Leff et al.: Sentiment alone will not be enough.

## Competing interests

The authors declare that they have no competing interests.

## Authors' contributions

GT conceived of the study, was involved in analyzing and interpreting the data and wrote the manuscript. MB was involved in study design, conducted the interviews, and performed the analysis of the data and interpretation of the data. CK, CM and EHP contributed to the interpretation of the data, the writing, and critical revision of the manuscript. All authors read and approved the final manuscript.

## Pre-publication history

The pre-publication history for this paper can be accessed here:

http://www.biomedcentral.com/1471-2296/12/24/prepub

## References

[B1] Kao H Conant R Soriano TMW The past, present, and future of house calls Clin Geriatr Med 2009 25 19 34 10.1016/j.cger.2008.10.005 19217490

[B2] Boerma WGW Groenewegen PP GP home visiting in 18 European countries. Adding the role of health system features Eur J Gen Pract 2001 7 132 7 10.3109/13814780109094331

[B3] Snijder EA Kersting M Theile G Kruschinski C Koschak J Hummers-Pradier E Junius-Walker U Home visits in German General Practice: findings from routinely collected computer data of 158,000 patients Gesundheitswesen 2007 69 679 85 10.1055/s-2007-993181 18181071

[B4] van den Berg M Cardol M Bongers FJM de Bakker D Changing patterns of home visiting in general practice: An analysis of electronical medical records BMC Fam Pract 2066 7 58 10.1186/1471-2296-7-58 PMC1624836 17044914

[B5] Aylin P Majeed A Cook DG Home visiting by general practitioners in England and Wales BMJ 1996 313 207 10 8696199 10.1136/bmj.313.7051.207 PMC2351617

[B6] Bass MJ McWhinney IR Stewart M Grindrod A Changing face of family practice. Trends from 1974 to 1994 in one Canadian city Can Fam Physician 1998 44 2143 9 9805169 PMC2277911

[B7] Bergeron R Laberge A Vezina L Aubin M Which physicians make home visits and why? A survey CMAJ 1999 161 4 369 74 10478159 PMC1230536

[B8] Oldenquist GW Scott L Finucane TE Home care: What a physician needs to know Clev Clin J Med 2001 68 5 433 40 10.3949/ccjm.68.5.433 11352323

[B9] Svab I Kravos A Vidmar G Factors influencing home visits in Slovenian general practice Fam Pract 2003 20 58 60 10.1093/fampra/20.1.58 12509372

[B10] De Maeseneer J De Prins L Heyerick JP Home visits in Belgium: A multivariate analysis Eur J Gen Pract 1999 5 1 11 4 10.3109/13814789909094245

[B11] Kersnik J Observational study of home visits in Slovene general practice: Patient characteristics, practice characteristics and health care utilization Fam Pract 2000 17 5 389 93 10.1093/fampra/17.5.389 11021897

[B12] Marcinowicz L Chlabicz S Gugnowski Z Home visits by family physicians in Poland: Patients' perspective Eur J Gen Pract 2007 13 237 45 10.1080/13814780701627362 18324506

[B13] Meyer GS Gibbons RV House calls to the elderly - a vanishing practice among physicians N Engl J Med 1997 337 1815 20 10.1056/NEJM199712183372507 9400040

[B14] Pereles L Home visits. An access to care for the 21st century Can Fam Physician 2000 46 2044 8 11072584 PMC2145106

[B15] Peppas G Theocharis G Karveli EA Falagas ME An analysis of patient house calls in the area of Attica, Greece BMC Health Serv Res 2006 6 112 10.1186/1472-6963-6-112 16953873 PMC1574301

[B16] Court BV Bradley CP Cheng KK Lancashire RJ Responding to out of hours request for visits: A survey of general practitioner opinion BMJ 1996 312 1401 2 8646100 10.1136/bmj.312.7043.1401a PMC2351107

[B17] Lattimer V Smith H Hungin P Glasper A George S Future provision of out of hours primary medical care: A survey with two general practitioner research networks BMJ 1996 312 352 6 8611835 10.1136/bmj.312.7027.352 PMC2350256

[B18] Van Royen P De Lepeleire J Maes R Home visits in general practice: An exploration by focus groups Arch Public Health 2002 60 371 84

[B19] Liang HW Landers S Who receives house calls? J Am Geriatr Soc 2008 56 1581 2 10.1111/j.1532-5415.2008.01782.x 18808612

[B20] Vinker S Nakar S Weingarten MA members of the Israeli General Practice Research Network Home visits to the housebound patient in family practice: A multicenter study IMAJ 2000 2 203 6 10774267

[B21] Caplan GA Ward JA Brennan NJ Coconis J Board N Brown A Hospital in the home: A randomised controlled trial Med J Australia 1999 170 156 60 10078179

[B22] Moraes E Campos GM Figlie NB Laranjeira R Ferraz MB Cost-effectiveness of home visits in the outpatient treatment of patients with alcohol dependence Eur Addict Res 2010 16 69 77 10.1159/000268107 20029212

[B23] Katz PR Karuza J Intrator O Mor V Nursing home physician specialists: A response to the workforce crisis in long-term care Ann Intern Med 2009 150 411 3 19293074 10.7326/0003-4819-150-6-200903170-00010 PMC4756641

[B24] Johnson MA Changing the culture of nursing homes Ann Intern Med 2010 170 5 407 9 10.1001/archinternmed.2009.550 20212175

[B25] Koopmans RTCM Lavrijsen JCM Dutch elderly care physicians: A new generation of nursing home physician specialists JAGS 2010 58 9 1807 8 10.1111/j.1532-5415.2010.03043.x 20863347

[B26] van den Berg N Meinke C Heymann R Fiß T Suckert E Pöller C Dreier A Rogalski H Karopka T Opperman R Hoffman W AGnES: Supporting general practitioners with qualified medical practice personnel. Model project evaluation regarding quality and acceptance Dtsch Ärztebl Int 2009 106 1-2 3 9 19564978 10.3238/arztebl.2009.0003 PMC2695315

[B27] Edwards M Bobb C Robinson SI Nurse practitioner management of acute in-hours home visit or assessment requests: A pilot study Br J Gen Pract 2009 59 7 11 10.3399/bjgp09X394798 19105910 PMC2605527

[B28] Leff B Burton JR The future history of home care and physician house calls in the United States J Gerontol 2001 56A 10 M603 8 10.1093/gerona/56.10.m603 11584032

